# Papillary Carcinoma in Struma Ovarii: A Radiological Dilemma

**DOI:** 10.7759/cureus.17360

**Published:** 2021-08-22

**Authors:** Joel J Thomas, Sagar Maheshwari, Mohammad Alwaheedy

**Affiliations:** 1 Radiology, Barking, Havering and Redbridge University Hospitals National Health Service (NHS) Trust, London, GBR

**Keywords:** struma ovary, rare tumour, ovarian tumour, papillary carcinoma, cystic mass, thyroid follicles, radiological dilemma, ectopic thyroid tissue, malignant struma ovarii

## Abstract

Struma ovarii is a very rare tumour of the ovary, which is usually benign. It is a solid cystic swelling of the ovary, which is characterised by the presence of histologically detectable thyroid tissue. Confirmatory diagnosis is usually on histopathology after resection of the tumour. It is difficult to identify struma ovarii with radiological investigations alone as it may mimic malignancy. In this case report, we present the case of a 48-year-old woman who had a total abdominal hysterectomy for an incidental finding of right adnexal mass on ultrasound scan following a 10-day history of loose stools and pain in the abdomen. It was ultimately found to be a follicular variant of papillary carcinoma in struma ovarii after a pathological examination.

## Introduction

Struma ovarii (SO) is a bizarre tumour of the ovary which is a form of matured ovarian teratoma. It is a variant of dermoid tumours of the ovary, in which the majority of the tissue components consist of thyroid tissue [1]. Although thyroid cells may be found in a small proportion of ovarian tumours, to qualify as an SO, thyroid proportion must constitute at least over 50% of the overall tissue [2].

This tumour was first described by Boëttlin in 1889 when he observed the presence of thyroid follicular tissue in the ovaries. It is usually benign but the malignant disease is found in a small proportion of the cases [3]. Reports show that patients with SO often would not manifest any symptoms or would present with symptoms similar to ovarian tumours that are non-specific in nature [4]. Certain authors have also reported ascites as a symptom in some of these patients. Hence, SO should be considered a differential in patients presenting with a pelvic mass, ascites, and elevated Ca-125 tumour marker. Confirmative diagnosis can be made only after the histological examination and surgical resection remain the mainstay modality of treatment.

## Case presentation

**Figure 1:**
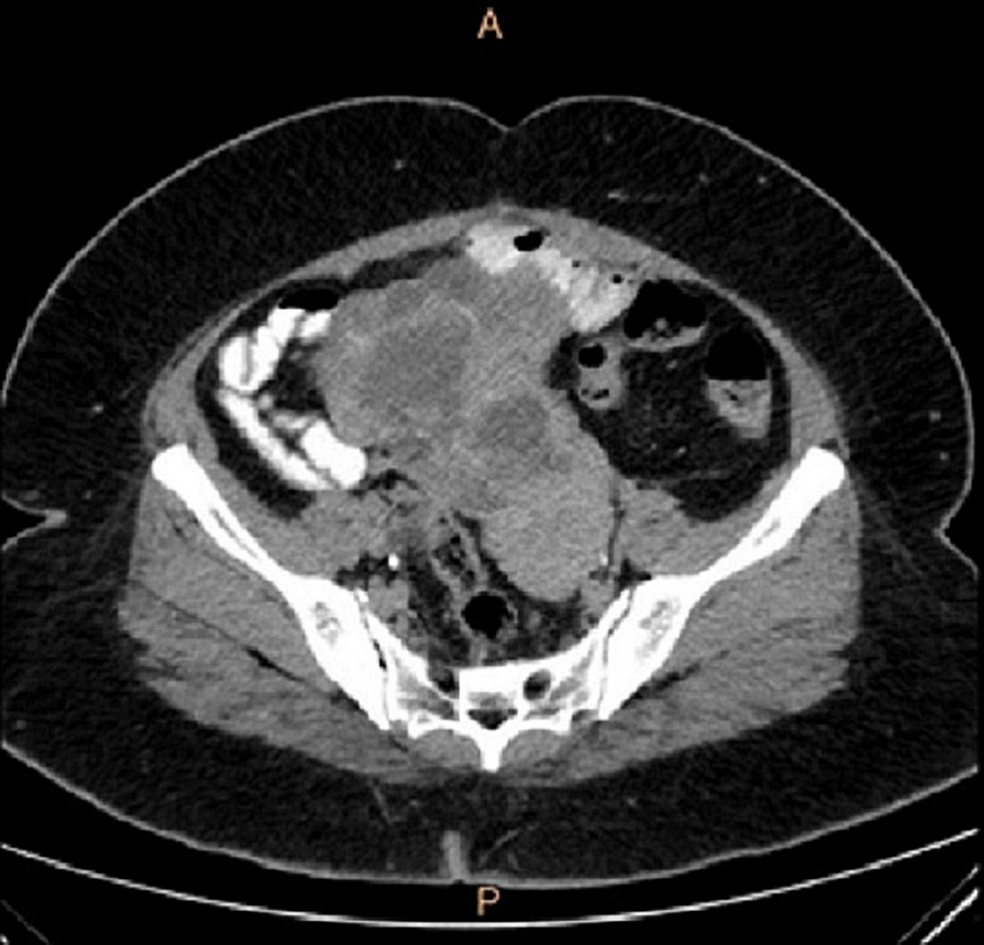
CT image showing lobulated heterogeneously enhancing complex cystic mass in the right adnexa

**Figure 2:**
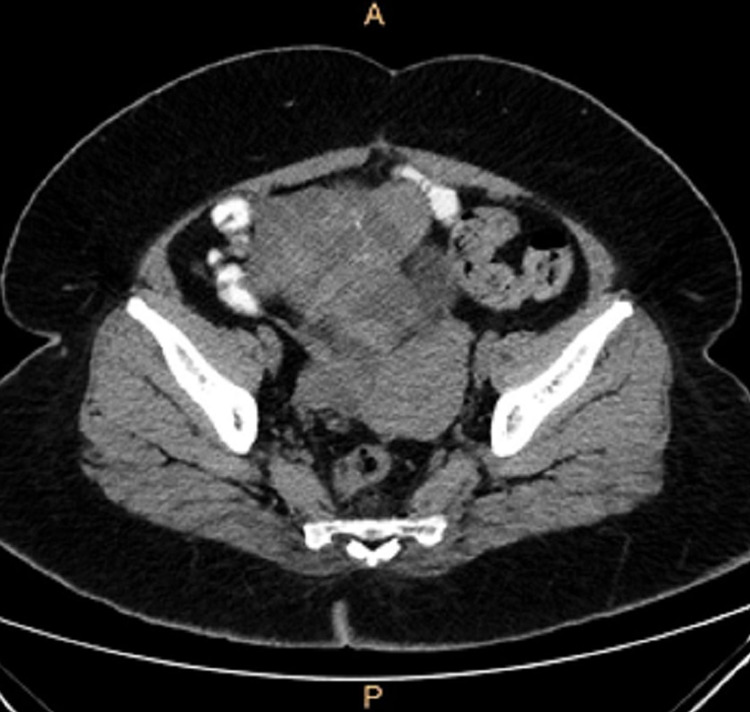
CT axial image - no fat density, calcification, no free fluid, lymphadenopathy and mesenteric nodules

A 48-year-old female (P2L2) was referred to our hospital for a CT scan after an ultrasound scan elsewhere, which revealed a cystic mass in the right adnexa with septations and solid components. She initially came to the hospital with complaints of loose watery stools for 10 days and lower abdominal pain for seven days. She was on day 10 of her menstrual cycle. She admitted to having regular cycles with normal flow. She was nondiabetic and normotensive. She had low-grade fever however no chills, no lower urinary tract symptoms or previous episodes of loose stools lasting for over three days. On examination, she had pallor and seemed lethargic, but had no icterus or lymphadenopathy. There was mild tachycardia (105/min) while the rest of her observations were stable. On abdominal examination, slight discomfort in the lower abdomen and pelvis region was noted; however, there was no obvious palpable mass. Ultrasound scan done elsewhere showed possible tubo-ovarian mass/abscess which could be infective or neoplastic. Apart from an elevated Ca-125 level (68U/L), all other blood investigations including thyroid profile came back normal. We initially considered a possibility of pelvic TB, but Mantoux test was negative. CT scan revealed lobulated heterogeneously enhancing complex cystic mass measuring approximately 9.2 x 6.8 x 4.3 cm in the right adnexa extending up to the pelvic brim (Figure 1). The right ovary could not be visualised separately from the lesion while the left ovary appeared normal. There were no areas of calcification or fat density noted. No free fluid could be visualised in the pelvis to suggest ascites (Figure 2). No significantly enlarged pelvic retroperitoneal lymph nodes were seen. The adrenal glands appeared to be normal and the adjacent bowels loops as well were unremarkable. No focal liver lesions were visible and the visualised lung segments did not contain any significant nodules. There were no destructive bony lesions noted. The scan also did not reveal omental caking or the presence of mesenteric nodules.

An ultrasound scan suggested the possibility of a tubo-ovarian mass or an infection, however, infection was unlikely after the CT scan. The absence of areas of calcification or fat density further ruled out the possibility of a mature cystic teratoma, while the presence of soft tissue component in the scan made mucinous cystadenoma a less likely differential. The characteristic feature of a large lobulated multiloculated solid cystic mass showing enhancement made malignant ovarian mass a strong possibility, although the absence of enlarged lymph nodes in the surrounding areas and lack of evidence of metastasis dampened the possibility.

**Figure 3:**
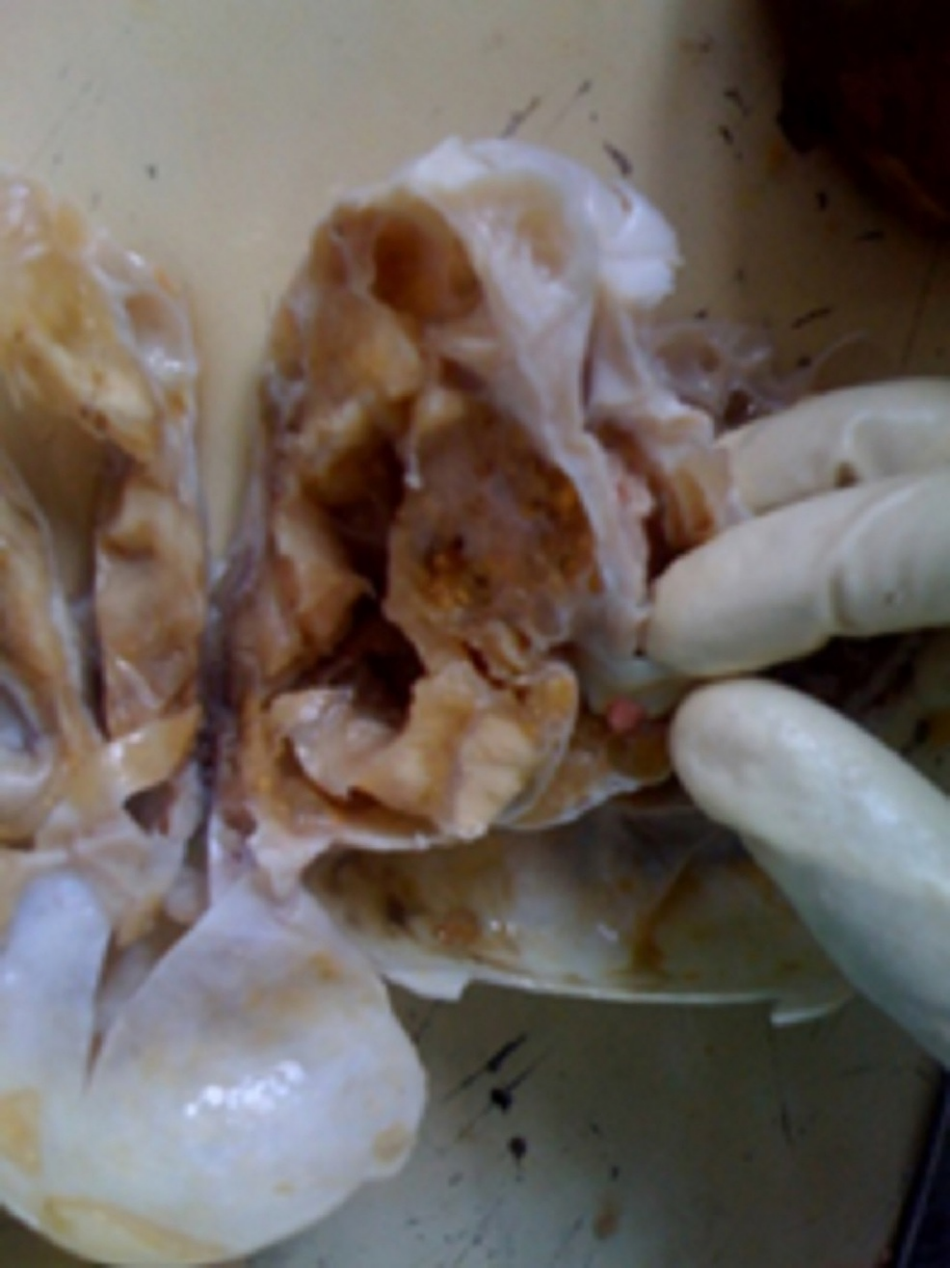
Specimen after resection of the tumour showing multiple cystic mass lesion

**Figure 4:**
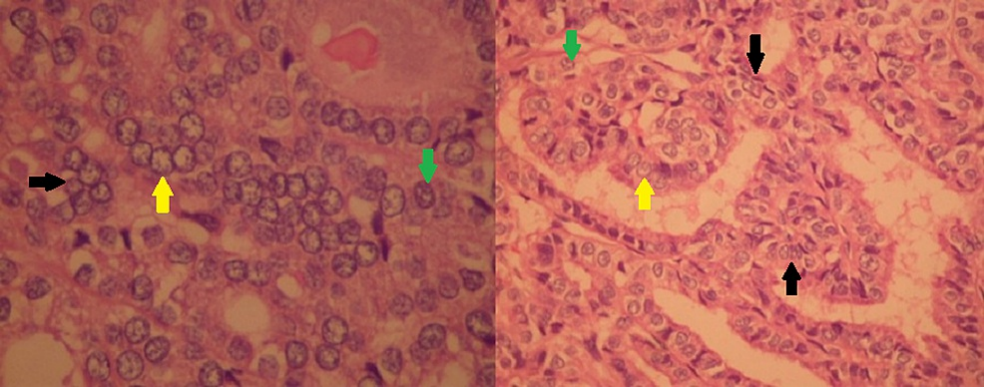
High magnified (left) and intermediate magnified (right) microscopic slides of the resected sample depicting papillary thyroid carcinoma in the ovary These cells clearly depict the characteristic enlarged and irregular nuclei with pale chromatin (yellow arrow). The black arrow represents crowding and overlapping nuclei. Some nuclei contain longitudinal grooves as a result of nuclear membrane infoldings and portray a characteristic coffee-bean appearance (green arrow).

The decision was taken for surgical resection of the tumour and confirmation of clinical diagnosis. The resected sample (Figure 3) was sent for histopathological examination and it was ultimately found to be follicular variant of papillary thyroid carcinoma in SO (Figure 4). Histology slides depict the characteristic ‘Orphan-Annie eye’ nuclei with pale chromatin and areas of nuclear overcrowding. Some nuclei also appear to be coffee-bean shaped due to nuclear membrane infoldings.

 

## Discussion

SO is commonly found in patients of their reproductive age. However, it can be seen in individuals of any age group including children [5]. It comprises a mere 1% of all ovarian tumours and 2.7% of all dermoid tumours, thereby making it a relatively rare tumour of the ovary [6]. Malignant transformation is uncommon in the struma ovary and metastasis is found in only about 5% of the malignant cases [1]. Papillary carcinoma is the most common variant among them; however, cases of follicular carcinoma are not so uncommon [7]. Most patients are asymptomatic clinically although some might present with abdominal pain, distention, urinary symptoms, hot flushes or infertility [1]. They are often diagnosed incidentally on physical examination or through sonography. They may sometimes be associated with ascites or both ascites and pleural effusion (pseudo-Meigs syndrome). Thyroid hormone levels may be elevated in some patients, which maybe because of the ectopic thyroid tissue in the ovary [8]. Ca-125 levels may be raised in patients with struma ovary even if they have a benign presentation. Gross macroscopic appearance is usually of a solid tumour but can sometimes also be solid-cystic or cystic, with mucous and gelatinous content [9]. On microscopy, it is composed of mature thyroid tissue with multiple follicles [8]. Ultrasound imaging characteristically confirms the presence of one or more ‘struma pearls’ in the ovary, which are well-defined solid tissue with a smooth outer surface. They closely resemble dermoid cysts, which are usually identified using an ultrasound as a ‘white ball’ containing hair and sebum. Vascularity on Doppler examination uniquely differentiates the struma ovary from a dermoid cyst [10]. In a study conducted by Sung II Jung, CT scans of 13 women pathologically diagnosed with struma ovary were evaluated retrospectively and he noticed that struma ovary typically appeared as multicystic masses with smooth margins. Precontrast scans detected high attenuation lesions while there were nil or minimal enhancement of the cyst wall [11]. MR imaging typically shows multilocular cystic lesions in which the signal intensities of the various components vary within loculi. Classical findings include low signal intensity on T1-weighted images and even lower intensity signals on T2-weighted images. This pattern of varying intensities may be attributed to the pathological finding of gelatinous colloid matter found in the large follicles [12]. These characteristic findings may not always be recognised on radiological examinations. Hence, histopathological diagnosis after surgical resection of the mass remains the mainstay in diagnosis. Unfortunately, many patients undergo unnecessary extensive resection in suspicion of malignancy [13].

## Conclusions

Papillary carcinoma of stuma ovarii is a very rare entity and is usually diagnosed post-operatively after histopathological examination of the resected sample. Here, we present the case of a 48-year-old woman who presented with pain in the abdomen and watery stools for seven days and was diagnosed to have papillary thyroid carcinoma in SO. She underwent a total abdominal hysterectomy after suspicion of malignancy from the elevated serum CA-125 levels and the presence of a lobulated complex cystic mass on CT. There are no definite guidelines for the management of this condition owing to its rarity; hence, therapeutic decisions should be made based on clinical judgement and histopathological reports.
